# Polymicrobial Infections Among Patients with Vascular Q Fever, France, 2004–2020

**DOI:** 10.3201/eid2707.210282

**Published:** 2021-07

**Authors:** Mathilde Puges, Xavier Bérard, Caroline Caradu, Maïlys Ducours, Carole Eldin, Mathilde Carrer, Noémie Sauvage, Marc-Olivier Vareil, Laure Alleman, Fatima M’Zali, Sabine Pereyre, Charles Cazanave

**Affiliations:** Centre Hospitalier Universitaire de Bordeaux, Bordeaux, France (M. Puges, X. Bérard, C. Caradu, M. Ducours, M. Carrer, N. Sauvage, S. Pereyre, C. Cazanave);; IHU-Méditerranée Infection, Marseille, France (C. Eldin);; Aix Marseille University, IRD, AP-HM, SSA, VITROME, Marseille (C. Eldin);; Centre Hospitalier de la Côte Basque, Bayonne, France (M.-O. Vareil, L. Alleman);; University of Bordeaux, Bordeaux (F. M’Zali, S. Pereyre, C. Cazanave)

**Keywords:** vascular Q fever, chronic Q fever, *Coxiella burnetii*, vascular graft and endograft infections, aortitis, aortoenteric fistula, Q fever, bacteria, zoonoses, bacterial zoonoses, vector-borne infections, polymicrobial infections, France

## Abstract

We report 5 cases of vascular Q fever complicated by polymicrobial superinfection in patients who had no risk factors for acute Q fever. Q fever was diagnosed by serologic and molecular assays for *Coxiella burnetii*. We confirmed additional infections using conventional graft cultures.

Vascular Q fever, which is caused by *Coxiella burnetii*, is well-described disease; recent publications on the topic include large studies in France and the Netherlands (*1*–*3*). Unlike other vascular graft and endograft infections, especially of aortic and aortoenteric fistulas, vascular Q fever is usually caused by a single microorganism. However, when clinical samples (e.g., blood, vascular graft tissue) test positive for bacterial infection, no recommendation exists for screening for additional microorganisms. Researchers have documented >5 cases of vascular Q fever complicated by polymicrobial superinfection, all involving a single co-infecting species: *Bacteroides fragilis*, *Streptococcus* spp., *S. anginosus*, *Yersinia enterocolitica*, or *Klebsiella pneumoniae* (*4*–*6*). Researchers also have documented several cases of Q fever endocarditis complicated by an additional microorganism: *Enterococcus faecalis*, *S. viridans*, *S. mitis*, *S. gallolyticus*, *S. salivarius*, *S. crispatus*, *S. gordonii*, or *Staphylococcus aureus* (*7*–*10*). We describe 5 cases of vascular Q fever complicated by polymicrobial superinfection.

## The Study

We retrospectively screened the Bordeaux University Hospital Vascular Infections database for patients with chronic Q fever treated at Bordeaux University Hospital (BUH; Bordeaux, France) or Bayonne District Hospital (Bayonne, France) during January 2004–June 2020. To be included in the study, patients had to have a *C. burnetii* phase I IgG titer >6,400 or molecular detection in blood or infected tissues, as well as clinical signs of a vascular infection or evidence from computed tomography or nuclear imaging scans (*6*). We conducted an immunofluorescence assay for *C. burnetii* at BUH and Bayonne Hospital, then sent the samples to the French National Reference Center (Marseille, France) for species confirmation (*6*). When patients had borderline or positive *C. burnetii* serologic results (phase I IgG titer >100), we also conducted PCR on an arterial biopsy or vascular graft sample. PCR also was conducted at the French National Reference Center as previously described (*6*). In accordance with national legislation, surviving patients did not object to the analysis of their data for research purposes.

Of 425 patients with vascular infections during January 2004–June 2020, 16 had Q fever, including 7 since 2019, when BUH and Bayonne Hospital began conducting systematic Q fever serologic assays for all patients with vascular infections. In total, 5 patients (1 with aortitis and 4 with vascular graft and endograft infections) had vascular Q fever complicated by polymicrobial superinfection in the abdominal aorta (Table 1). Of the 5 cases, 4 had occurred since 2015. All 5 patients had undergone surgery; 4 had an aortoduodenal fistula, and the remainder had intimate contact between the aortic graft and the duodenum.

**Table 1:** Characteristics of patients with vascular Q fever complicated by polymicrobial infections, France, 2014–2020*

Patient no.	Age, y	Infection type	Graft type (age, mo)	Symptoms	Computed tomography scan findings	Phase I titer, IgG/IgM/IgA	*Coxiella burnetii* PCR	AEF	Replacement graft type	Co-infections	Clinical outcome
1	64	Aortitis	Not available	Hemorrhagic shock caused by lower gastrointestinal bleeding	Abdominal aortic aneurysm, psoas hematoma, AEF	800/0/400	Aortic graft, +	AD	Bovine pericardium	Aorta: *Enterobacter cloacae*, *Enterococcus faecalis*, *Enterococcus durans*, *Streptococcus anginosus*, *S. salivarius*, *Klebsiella oxytoca*, *Gemella haemolysans*, *S. mitis*, *Escherichia coli*, *Haemophilus parainfluenzae*; blood culture, –	Survived; completed 18-mo Q fever treatment 28 d after symptom onset; has maintained remission 5 y after diagnosis
2	78	Aortic endograft	Polyester endograft (96)	Chills, back pain, acute respiratory failure, fever	Periprosthetic abscess and gas, AEF	100/0/0	Aortic graft, +; serum, –	No, but AD close contact	Silver and triclosan impregnated graft	Graft: *S. anginosus*, *Actinomyces odontolyticus*, *S. oralis*; blood culture: *S. anginosus*	Died 37 d after multiple organ failure caused by PVGI; did not receive Q fever treatment
3	63	Aortic endograft	Polyester endograft (72)	Skin infection, calf abscess, fever	Close contact with duodenum	Borderline	Aortic graft, +	AD	Bovine pericardium	Graft: *E. cloacae*, *Candida albicans*; blood culture: *E. cloacae*	Died of unrelated causes; began Q fever treatment 11 d after symptom onset; attained remission at 18 mos; died at 19 mos.
4	80	Vascular endograft	Polyester endograft (24)	Abdominal pain, septic shock, fever	Periprosthetic gas	1,600/0/800	Aortic graft, +; serum, –	AD	Silver and triclosan impregnated graft, stent	Graft: *A. odontolyticus*, *S. gordonii*, *S. oralis*, *Eikenella corrodens*; blood culture: *Granulicatella adiacens*, *Veillonella parvula*, *S. gordonii*	Died on day 27 of hemorrhagic shock on a recurrent AEF caused by PVGI; began Q fever treatment 20 d after symptom onset.
5	70	Aortic graft	Silver polyester graft (36)	Back pain, spondylodiscitis, lower gastrointestinal bleeding, fever	Periprosthetic infiltration, spondylodiscitis	400/0/100	Vertebral biopsy, +	AD	Silver impregnated polyester graft	Graft: *Hafnia alvei*, *E. coli*, nonhemolytic *Streptococcus* spp., *Lactobacillus* spp.; blood culture, –	Died on day 173 after symptom onset, 12 d after hospitalization for multiple organ failure caused by PVGI; did not receive Q fever treatment.

*AD, aortoduodenal; AEF, aortoenteric fistula; PVGI, prosthetic vascular graft infection; +, positive; –, negative.

In total, 4 patients had *C. burnetii* phase I IgG titers >100 and <6,400. Patient 3 had a borderline result; therefore, that patient’s sample was not sent to the French National Reference Center for species confirmation (Tables 1,2). Four patients tested positive by PCR on vascular or graft samples, whereas patient 5 tested positive by PCR on a vertebral biopsy (Table 1). We conducted PCR on serum samples from 2 patients; the samples tested negative for *C. burnetii*. None of the patients had risk factors for acute Q fever, such as contact with animals, consumption of raw milk, or tick bites. Three of the patients lived in the countryside of the Nouvelle-Aquitaine region. We isolated 2–10 additional microorganisms using conventional graft cultures, identifying concurrent bacteremia in 3 patients (patients 2, 3, and 4) (Table 1). All isolated microorganisms were common commensals of the oral or gut microflora. Only patient 3 had a fungal co-infection (*Candida albicans*).

**Table 2:** Serologic assay results of patients with vascular Q fever complicated by polymicrobial infections, France, 2014–2020*

Serologic results, time of assay†	Patient (month of Q fever diagnosis)				
	1 (2015 Apr)	2 (2020 Jun)	3 (2015 Jul)	4 (2019 Sep)	5 (2010 Dec)
Before Q fever diagnosis	NA	NA	NA	NA	2007 Aug 9: 1,600/0/0–3,200/0/0; 2007 Aug 29: 1,600/0/0–3,200/0/0; 2007 Nov: 1,600/0/0–3,200/0/0; 2008 May: 800/0/0–1,600/0/0
At Q fever diagnosis	2015 Apr: 800/0/200–100/0/0	2020 Jun 15: 100/0/0–100/0/0; 2020 Jun 17: 100/0/0–100/0/0	Borderline	2019 Sep: 1,600/0/800–1,600/0/800	2010 Dec: 400/0/100–800/0/200
After Q fever diagnosis	2015 Jul: 800/0/200–400/0/0; 2016 Aug: 800/0/400–200/0/0; 2017 Dec: 400/0/200–200/0/100	NA‡	NA	NA‡	NA‡

*NA, not available. †Values are titers against *C. burnetti* phase I–phase II (IgG/IgM/IgA). ‡Values NA because of patient death: patient 2 died on day 37, patient 4 died on day 27, and patient 5 died on day 12.

Patients 3 and 5 had been treated for previous episodes of vascular Q fever; their infections relapsed after the end of treatment. Patient 3 had been treated with hydroxychloroquine and doxycycline for 2 years for a Q fever aortic graft infection. The infected graft was not removed, and the infection relapsed 2 months after the end of treatment. Patient 5 had Q fever aortitis and spondylodiscitis 4 years before this episode. He had been treated with hydroxychloroquine and doxycycline for 18 months and had received an aortic graft implantation. However, the infection in the aortic graft relapsed 2 years after the end of treatment.

In total, 3 patients died of vascular graft and endograft infections; another died of a different cause. Patients 1, 3, and 4 were treated with hydroxychloroquine and doxycycline. Only patients 1 and 3 completed the 18-month therapy; the other patients died before or during treatment (Table 1). All patients also had a 6-week course of antimicrobial therapy for the other identified microorganisms.

## Conclusions

We identified 5 cases of vascular Q fever complicated by polymicrobial superinfection in patients with no documented risk factors for acute Q fever. Q fever was diagnosed by serologic and molecular assays. We isolated additional microorganisms from clinical samples from 5 of 16 patients with vascular fever, suggesting that co-infections might be more common than previously thought. Furthermore, 4 of the cases complicated by polymicrobial superinfection were diagnosed during the past 5 years, suggesting that this condition might be emergent.

All patients had an aortoduodenal fistula or intimate contact between the aortic graft and the duodenum. The role of *C. burnetii* in vascular fistulas is well-described, especially in aortoenteric fistulas but also in aortobronchial, aortocaval, and arteriocutaneous fistulas (*2*,*11*–*13*). Death rates among patients with chronic Q fever complicated by arterial fistula are higher than among those without fistulas (*2*). Aortoenteric fistulas arise from infection and inflammation of the aortic wall or the perigraft tissues created by *C. burnetii* infection, which erodes the adjacent digestive tract. Diagnostic delays might contribute to fistula development; therefore, earlier detection of vascular Q fever might reduce the incidence of these complications.

We found that 31% of patients with vascular Q fever in this study also had an aortoenteric fistula, a concurrent condition that might have contributed to superinfection. This rate is higher than that suggested by previously published studies on vascular Q fever and aortoenteric fistulas (*2*). We might have found a higher rate because we have conducted systematic Q fever screening in every patient with vascular infection since 2019.

In conclusion, we report a small case series of vascular Q fever complicated by polymicrobial superinfection. Our findings support systematic screening for *C. burnetii* in patients with vascular infections, especially when an arterial fistula is suspected or confirmed. We believe these screenings should be conducted even when more common microorganisms are isolated by culture. The screening should not be limited to patients with risk factors for acute Q fever. The patients in this study had low *C. burnetti* phase I IgG titers (none >6,400) and all had vascular Q fever confirmed by molecular diagnosis. Low phase I IgG titers have been described in acute Q fever endocarditis (*10*,*14*), suggesting that some of our patients might have had acute rather than chronic vascular Q fever. Therefore, physicians should conduct PCR selective for *C. burnetii* on vascular grafts or arterial biopsies when patients with a vascular infection have a phase I IgG titer >100. However, this low cutoff might impair specificity and positive predictive value (*15*) and should be further investigated. We highlight that vascular Q fever requires a specific and prolonged therapy, including surgery, to prevent relapse and other complications. We emphasize the need for systematic *C. burnetii* screening in patients with vascular infections, even when cultures test positive for other microbes.
